# Scoring of medial arterial calcification predicts cardiovascular events and mortality after kidney transplantation

**DOI:** 10.1111/joim.13459

**Published:** 2022-02-11

**Authors:** Helen Erlandsson, Abdul Rashid Qureshi, Jonaz Ripsweden, Ida Haugen Löfman, Magnus Söderberg, Lars Wennberg, Torbjörn Lundgren, Annette Bruchfeld, Torkel B. Brismar, Peter Stenvinkel

**Affiliations:** ^1^ Division of Transplantation Surgery Department of Clinical Science Intervention and Technology Karolinska University Hospital Karolinska Institutet Stockholm Sweden; ^2^ Department of Health Medicine and Caring Sciences Linköping University, Linköping Sweden; ^3^ Division of Renal Medicine Department of Clinical Science Intervention and Technology Karolinska Institutet Stockholm Sweden; ^4^ Department of Radiology Karolinska University Hospital Stockholm Sweden; ^5^ Unit of radiology, Department of Clinical Science Intervention and Technology Karolinska Institutet Stockholm Sweden; ^6^ Section of Cardiology Department of Medicine Karolinska Institutet Karolinska University Hospital Stockholm Sweden; ^7^ Cardiovascular, Renal and Metabolism Safety Clinical Pharmacology and Safety Sciences R&D, AstraZeneca Gothenburg Sweden

**Keywords:** cardiovascular events, coronary artery calcification, kidney failure, medial calcification, mortality

## Abstract

**Background:**

Progression of vascular calcification causes cardiovascular disease, which is the most common cause of death in chronic kidney failure and after kidney transplantation (KT). The prognostic impact of the extent of medial vascular calcification at KT is unknown.

**Methods:**

In this prospective cohort study, we investigated the impact of medial calcification compared to a mix of intimal and medial calcification represented by coronary artery calcification (CAC score) and aortic valve calcification in 342 patients starting on kidney failure replacement therapy. The primary outcomes were cardiovascular events (CVE) and death. The median follow‐up time was 6.4 years (interquartile range 3.7–9.6 years). Exposure was CAC score and arteria epigastrica medial calcification scored as none, mild, moderate, or severe by a pathologist at time of KT (*n* = 200). We divided the patients according to kidney failure replacement therapy during follow‐up, that is, living donor KT, deceased donor KT, or dialysis.

**Results:**

Moderate to severe medial calcification in the arteria epigastrica was associated with higher mortality (*p* = 0.001), and the hazard ratio for CVE was 3.1 (95% confidence interval [CI] 1.12–9.02, *p* < 0.05) compared to no or mild medial calcification. The hazard ratio for 10‐year mortality in the dialysis group was 33.6 (95% CI, 10.0–113.0, *p* < 0.001) compared to living donor recipients, independent of Framingham risk score and prevalent CAC.

**Conclusion:**

Scoring of medial calcification in the arteria epigastrica identified living donor recipients as having 3.1 times higher risk of CVE, independent of traditional risk factors. The medial calcification score could be a reliable method to identify patients with high and low risk of CVE and mortality following KT.

## Graphical abstract

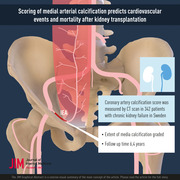

**Abbreviations**
AU: Agatston unit
AVC: aorta valve calcification
AMI: acute myocardial infarction
BMI: body mass index
CAC: coronary artery calcification
CV: cardiovascular
CVD: cardiovascular disease
CKD: chronic kidney disease
eGFR: estimated glomerular filtration rate
FRS: Framingham risk score
HR: hazard ratio
IQR: interquartile range
KFRT: kidney failure replacement therapy
KT: kidney transplantation
DDKT: deceased donor kidney transplant recipients
LDKT: living donor kidney transplant recipient
MC: medial calcification
VC: vascular calcification
PEW: protein energy wasting

## Introduction

Cardiovascular disease (CVD) is the leading cause of death among patients with chronic kidney disease (CKD) [1, 2]. CKD patients are exposed to a process of early vascular ageing characterized by endothelial dysfunction and vascular calcification (VC) leading to increased vascular stiffness and CVD [3]. The cause(s) of progression of VC in kidney failure replacement therapy (KFRT) is multifactorial, consisting of both traditional and nontraditional risk factors. Traditional risk factors include hypertension, smoking, hyperlipidemia, diabetes mellitus, age, and male sex [4]. Chronic kidney disease–mineral and bone disorder with abnormal calcium and phosphorus levels, chronic inflammation, the uremic milieu, oxidative stress, and down‐regulation of inhibitors of calcification are some of the nontraditional causes. These factors together with disruption of calcium and phosphate homeostasis in the uremic milieu drive extra‐osseous calcification via differentiation of vascular smooth muscle cells into osteoblast‐like cells [5, 6, 7, 8]. Patients who undergo kidney transplantation (KT) have lower risk for cardiovascular events (CVE) and mortality compared to patients remaining on dialysis and staying on the waitlist [9, 10, 11]. Still, the risk for CVE after KT is higher than in the general population and it is the leading cause of death and graft loss [12, 13]. In previous studies, assessment of coronary artery calcification (CAC) by CT has been used as a surrogate marker of the extent of VC because it is associated with an increased risk of CVE, heart failure, renal function decline, and mortality in CKD patients [14, 15, 16, 17]. CAC represents a mix of intimal and medial calcification. Atherosclerotic (intimal) and arteriosclerotic (medial) calcification appear to result in different cardiovascular (CV) insults and risk factor profile [18, 19]. Intimal atherosclerosis mainly affects elastic vessels, such as the aorta and the descendent branches, and is associated with inflammation and typical risk factors for atherosclerotic disease, such as diabetes mellitus, hypertension, smoking, and dyslipidemia. Because intimal atherosclerosis causes plaque instability, it increases the risk of acute coronary syndrome or acute myocardial infarction (AMI). Medial calcification (MC) is typically seen in diabetes and CKD, and it seems to be induced by a shift in vascular smooth muscle cell phenotype [20]. Inflammation and oxidative stress under the effect of uremic toxins contribute to this shift. Increased MC results in arterial stiffness and increased pulse pressure, which augments the risk of left ventricular hypertrophy, cardiac failure, and chronic peripheral vascular disease [21, 22]. Pulse wave velocity (PWV) can be used to evaluate the initial stiffening of the arterial wall, often due to medial calcification. The role of PWV in risk prediction in CKD needs to be investigated in larger randomized studies to determine the potential beneficial role in clinical practice [23, 24].

Because it is difficult to obtain arterial biopsies in routine clinical practice, a clear‐cut distinction between intimal and medial calcification has not been possible [22]. Measurements of calcification in the carotid artery by ultrasound, computed tomography (CT) scan of coronary arteries or abdominal aorta, and visual assessment on radiographs and mammographies have previously been used to examine the degree of arterial medial and intimal calcification in CKD patients [25, 26]. However, current radiological techniques do not allow a distinct separation of the two types of calcification. Thus, investigation and comparisons of the two different types of calcification and the effect on CVE in kidney failure is an underinvestigated topic. The aim of this prospective study was to determine whether scoring of MC has additional value in assessing intimal and medial calcification when predicting CVE and mortality after KT.

## Methods

**Fig. 1:**
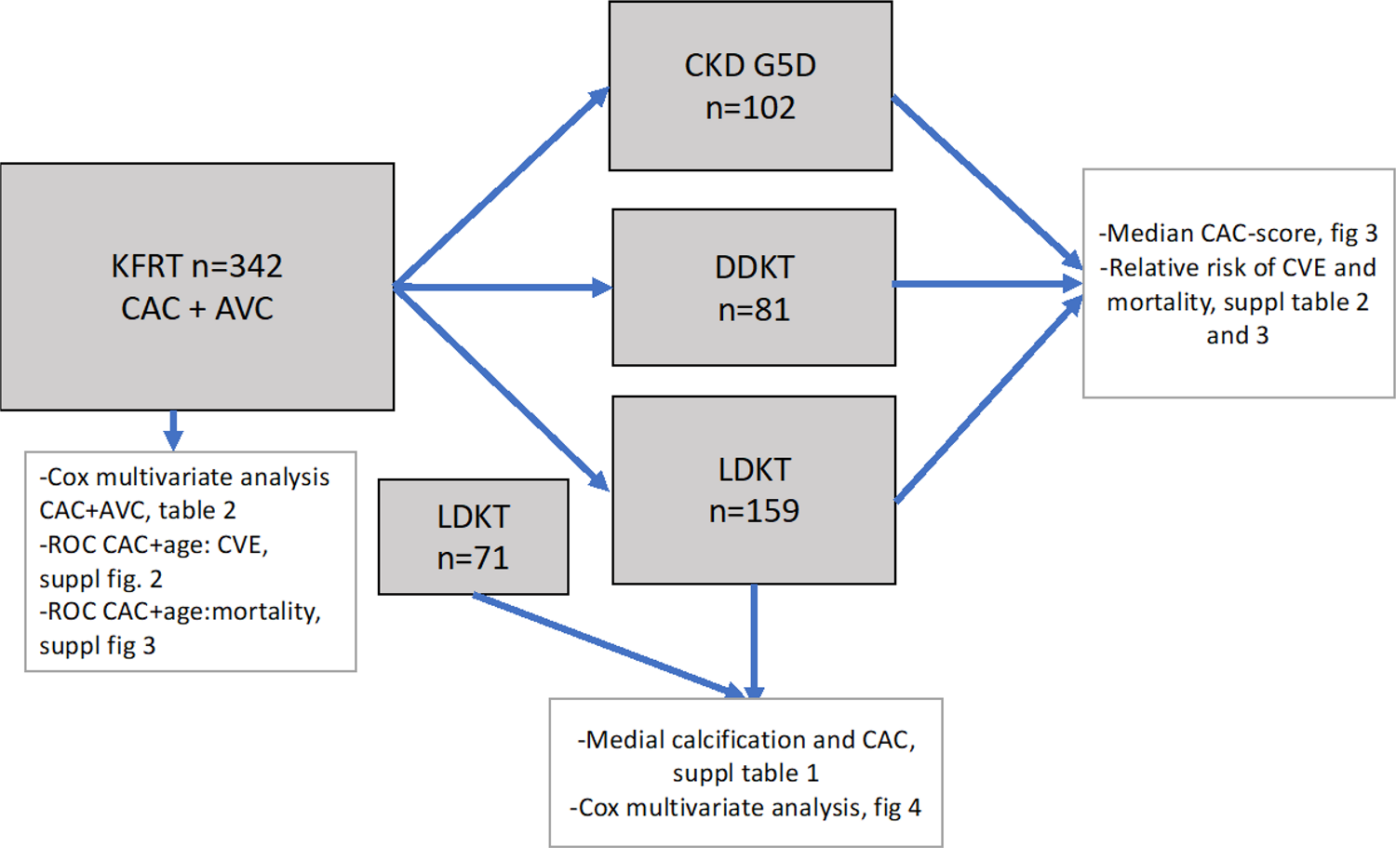
Schematic study protocol.

**Fig. 2:**
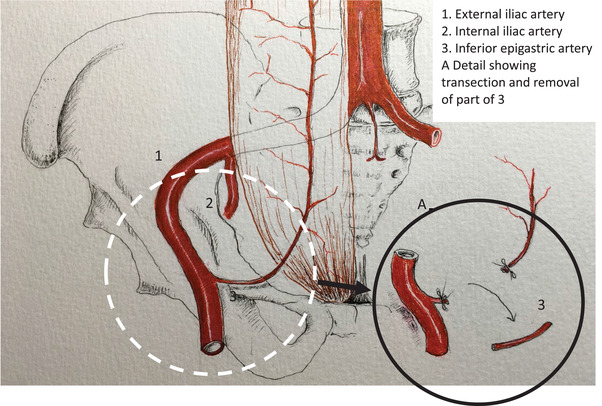
Biopsy of inferior epigastric artery during transplantation. The inferior epigastric artery is routinely transected at kidney transplantation to facilitate access to the urinary bladder. A part of the artery is taken for research as shown in the figure. Drawn by courtesy of Dr. John Sandberg.

In this prospective cohort study with 342 KFRT patients, we measured CAC score and aortic valve calcification (AVC) at time of KT or start of dialysis to investigate their long‐term predictive value (Fig. 1). In 102 Chronic Kidney Disease Group 5 Dialysis (CKD G5D) patients, KT was not performed during the observation period. The patients remained on dialysis due to comorbid conditions or were accepted to the waitlist but not transplanted. In the remaining patients, deceased donor kidney transplantation (DDKT, *n* = 81) and living donor kidney transplantation (LDKT, *n* = 159) were performed. The primary outcome was CVE and mortality. In 200 LDKT patients (including the 159 patients with measured CAC score + AVC), biopsies of the arteria epigastrica were retrieved intraoperatively (Fig. 2). The extent of MC in the epigastric artery was graded semiquantitatively as 0 (none), 1 (mild), 2 (moderate), or 3 (extensive) by an experienced pathologist (M.S.). The microscopic evaluation was performed using the von Kossa histochemical stain (Fig. S1). In the following analyses, we stratified calcification scoring in two groups: 0+1 (low grade) and 2+3 (high grade), respectively. Patients were included in the study during the period from March 2, 2006 to February 10, 2020, in Stockholm. Follow‐up time was up to 10 years (median 6.4 years, interquartile range [IQR] 3.7–9.6 years). To reduce selection bias, there were no exclusion criteria in the study. Only patients who did not want to participate or were in a physical condition that made computed tomography (CT) impossible were excluded. CT of coronary arteries and the aortic valve was performed at baseline in all patients to investigate prevalent CAC and AVC. AVC and CAC Agatston scores were calculated from noncontrast multidetector cardiac CT scans (LightSpeed VCT or Revolution CT; GE Healthcare, Milwaukee, WI, USA) using a standard ECG‐gated protocol and semi‐automatic software (syngo.via CT CaScoring; Siemens Healthcare, Forchheim, Germany). CAC and AVC were assessed from lesions with an area >1 mm^2^ and peak intensity >130 Hounsfield units (HU) based on the Agatston method and expressed in Agatston units (AU) [27]. Total CAC score was calculated as the sum of the CAC scores in the left main artery, the left anterior descending artery, the left circumflex artery, and the right coronary artery. AVC was determined as the sum of total calcifications in the aortic valve area, including calcifications within the valve leaflets as well as in the aortic wall immediately connected to the leaflets. Information concerning traditional CV risk factors—including Framingham score—and nontraditional risk factors—such as handgrip‐strength, plasma‐albumin, protein energy wasting (PEW), highly sensitive CRP (hsCRP), interleukin‐6 (IL‐6), calcium, phosphate, and intact parathyroid hormone (iPTH)—were collected at baseline. Clinical outcome data and date of mortality were retrieved from patient files. CVE were defined as either of the following occurring after inclusion: myocardial infarction (non‐ST‐elevated myocardial infarction, AMI), onset of ischemic heart disease requiring PCI, stroke, transitory ischemic attack, peripheral vascular ischemia, and severe aortic valve stenosis requiring surgery. Three patients were lost to follow‐up due to moving abroad, and they were censored. The study was conducted in adherence to the Declaration of Helsinki and approved by the Swedish Ethical Review Authority. The clinical and research activities reported are consistent with the Principles of the Declaration of Istanbul as outlined in the “Declaration of Istanbul on Organ Trafficking and Transplant Tourism.” Written informed consent was obtained from each patient.

### Statistical analysis

Data are expressed as median (10–90th percentile) or percentage. Statistical significance was set at the level of *p* < 0.05. Comparisons between two groups were assessed with the nonparametric Wilcoxon test for continuous variables and chi‐square test for nominal variables. Comparisons between two groups were assessed using the nonparametric Wilcoxon test for skewed continuous variables, Student's *t*‐test for normally distributed variables, and chi‐square test and Fisher's exact test for nominal variables. Kaplan–Meier survival curves were used to analyze univariate models. Patients were censored at 10 years. The predictors of all‐cause mortality and CVE were calculated by the area under curve by the receiver operating characteristic (ROC) curve. Multivariate Cox proportional hazard models were used for all‐cause mortality and CVE to obtain hazard ratios for one standard deviation (1 SD) increase of hsCRP and Framingham risk score (FRS). Discriminative abilities of the models were estimated as C‐statistics for Cox regression models [28]. To enable adjustment for confounders, we performed multivariate Cox analyses and included age, sex, diabetes mellitus, FRS, hsCRP, PEW, and treatment modality (LDKT, DDKT, and CKD G5D) in the different models. To further examine and compare the subgroups (LDKT, DDKT, and CKD G5D), we performed an analysis of baseline CAC in each group to investigate baseline differences.

Statistical analyses were performed using the statistical software SAS version 9.4 (SAS Campus Drive, Cary, NC, USA) and Stata 17.0 (Stata Corporation, College Station, TX, USA).

## Results

### CAC score, AVC, CV events, and mortality

**Fig. 3:**
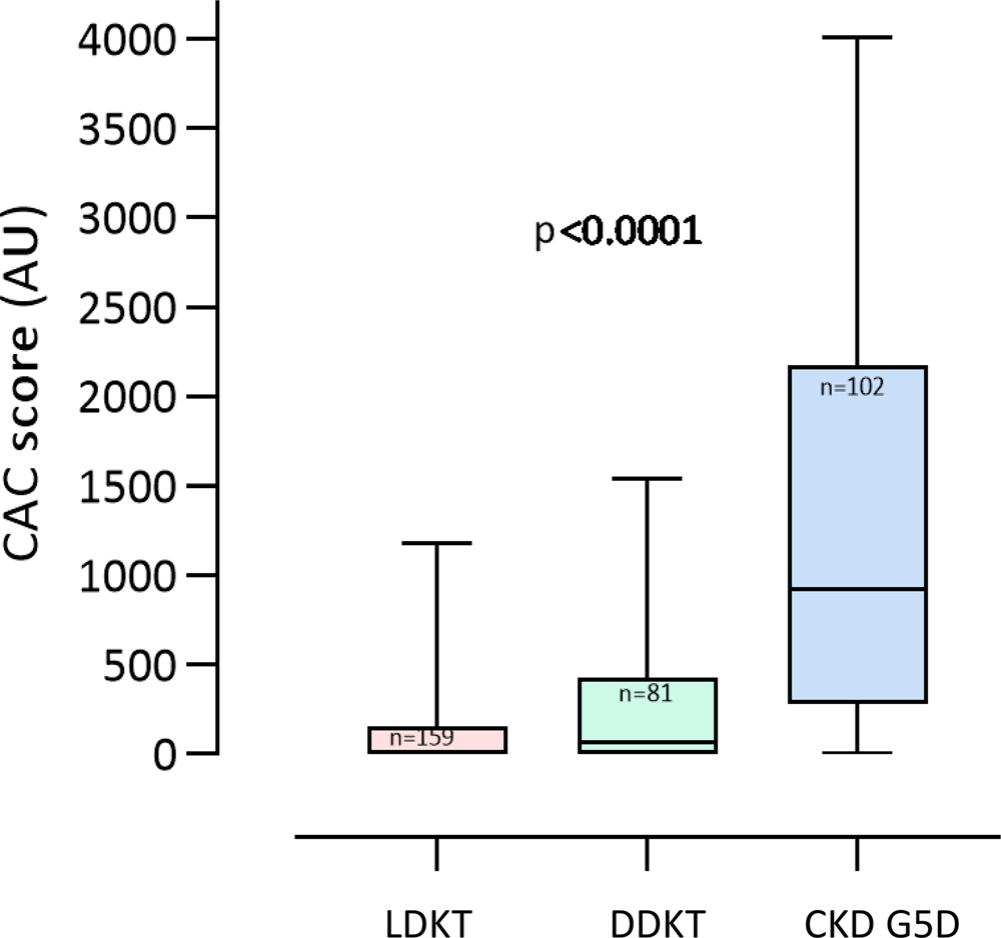
Comparison of baseline median and interquartile range (IQR) coronary artery calcium (CAC) score in patients receiving living donor allograft (LDKT), deceased donor allograft (DDKT), and patients remaining in dialysis (CKD G5D).

CAC and AVC were measured in 342 KFRT patients (median age 53 years, 66% males and 17% diabetics) (Table 1). In univariate analysis, prevalent CAC at baseline was associated with age, male sex, diabetes mellitus, CVD, lower diastolic blood‐pressure, estimated glomerular filtration rate (eGFR), PEW, body mass index (BMI), FRS, lower hand grip strength, lower albumin, hsCRP, IL‐6, and AVC (Table 1). In Cox regression analysis, CAC was associated with a higher risk of CVE independent of FRS, dialysis treatment at baseline, inflammation, and PEW (hazard ratio [HR] 2.5, 95% CI, 1.6–4.1; *p* < 0.001) (Table 2). AVC was associated with a higher HR for CVE in the same analysis (HR 2.8, CI 95% 1.2–6.9; *p* = 0.023). When baseline CAC score was compared in the three subgroups of KFRT patients (LDKT, DDKT, and CKD G5D), significant differences in median CAC score were observed: 3 AU (IQR 0–150) in LDKT, 65 AU (IQR 0–422) in DDKT, and 847 AU (IQR 306–2168) in CKD G5D, respectively (Fig. 3).

**Table 1:** Baseline characteristics in 342 KFRT patients according to the presence of CAC

	Total	Ref CAC <0	CAC 1–200	CAC 201–400	CAC >401	
	N = 342	N = 111	N = 87	N = 24	N = 120	*p*‐Value
Age, years	53 (42–65)	35 (27–49)	50 (43–57)	61 (54–69)	65 (57–72)	<0.001
Male sex, *n* (%)	227 (66.4%)	69 (62.2%)	54 (62.1%)	13 (54.2%)	91 (75.8%)	0.046
Diabetes mellitus, *n* (%)	55 (16.9%)	5 (4.8%)	7 (8.3%)	3 (13.0%)	40 (35.4%)	<0.001
CVD, *n* (%)	64 (19.5%)	7 (6.6%)	11 (13.1%)	3 (13.0%)	43 (37.4%)	<0.001
Systolic BP, mm Hg	144 (130–157)	142 (127–153)	144 (134–161)	144 (128–150)	144 (132–163)	0.19
Diastolic BP, mm Hg	85 (77–94)	89 (79–96)	88 (78–95)	84 (74–92)	81 (76–90)	0.002
eGFR ml/min/1.73 m^2^ (Epi)	6.1 (5.1–7.8)	6.8 (5.3–8.5)	5.9 (4.7–8.3)	6.2 (4.9–6.8)	5.8 (4.8–7.3)	0.028
Malnutrition PEW (SGA)	119 (36.5%)	46 (42.6%)	18 (21.7%)	7 (29.2%)	48 (43.2%)	0.006
BMI, kg/m^2^	24.8 (22.6–27.8)	23.5 (21.1–26.1)	25.1 (23.2–28.6)	25.7 (23.1–27.6)	25.3 (23.4–28.6)	<0.001
Framingham risk score	13.0 (5.2–25.1)	4.1 (1.5–8.9)	10.2 (5.6–17.3)	20.4 (7.9–26.1)	27.8 (16.5–42.8)	<0.001
Hand grip strength, %	85.6 (69.8–102.3)	96.3 (74.4–107.4)	93.0 (79.3–107.6)	84.5 (59.0–95.3)	74.4 (63.0–86.0)	<0.001
Hemoglobin, g/L	112 (104–120)	108 (99–119)	114 (107–123)	114 (110–128)	112 (104–119)	0.035
Albumin, g/L	34 (31–37)	35 (32–38)	35 (32–38)	33 (31–36)	32 (29–36)	<0.001
Triglyceride, mmol/L	1.5 (1.1–2.1)	1.4 (1.1–1.9)	1.5 (1.2–1.9)	1.5 (0.9–2.5)	1.6 (1.2–2.2)	0.29
Total cholesterol, mmol/L	4.4 (3.7–5.2)	4.4 (3.8–5.1)	4.4 (3.8–5.3)	4.7 (3.7–5.8)	4.4 (3.6–5.0)	0.53
Calcium, mmol/L	2.3 (2.1–2.4)	2.3 (2.1–2.4)	2.3 (2.2–2.4)	2.3 (2.2–2.4)	2.3 (2.1–2.4)	0.69
Phosphate, mmol/L	1.8 (1.5–2.1)	1.7 (1.4–2.0)	1.8 (1.5–2.1)	1.6 (1.4–1.8)	1.8 (1.5–2.3)	0.067
iPTH, ng/L	259 (160–426)	235 (141–415)	315 (182–456)	259 (94–348)	270 (168–392)	0.18
hsCRP, mg/L	3.4 (1.0–9.0)	1.8 (1.0–8.0)	2.0 (1.0–4.0)	5.8 (4.6–9.0)	4.4 (1.3–11.0)	0.021
IL‐6, pg/ml	3.5 (1.7–7.4)	1.9 (0.6–3.2)	2.3 (0.9–5.7)	5.5 (2.1–8.8)	5.8 (3.5–9.5)	<0.001
CAC Score (AU)	74 (0–871)	0 (0–0)	40 (11–91)	326 (276–353)	1432 (761–2473)	<0.001
CAC volume (mm^3^)	59 (0–652)	0 (0–0)	33 (10–67)	245 (208–271)	1121 (599–1953)	<0.001
Aorta score (AU)	0 (0–24)	0 (0–0)	0 (0–0)	0 (0–39)	33 (0–127)	<0.001

*Note*: Data are presented as median (IQR, interquartile range) for continuous measures, and *n* (%) for categorical measures.

Abbreviations: AU, Agatston units; AVC, aortic valve calcium; BMI, body mass index; CAC, coronary artery calcium; CVD, cardiovascular disease; DBP, diastolic blood pressure; %HGS, hand grip strength, converted to % of sex‐matched healthy controls; hsCRP, high sensitivity C‐reactive protein; IL‐6, interleukin‐6; iPTH, intact parathyroid hormone; PEW, protein‐energy wasting; SBP, systolic blood pressure; SGA, subjective global assessment.

**Table 2:** Cox regression in the presence of aortic calcium score (AVC > 0), coronary artery calcium (CAC > 0), Framingham risk score, inflammation (1‐SD hsCRP), subjective global nutritional assessment, and CVD events n = 310

_t	HR	SE	*z*	*P* > |*z*|	95% CI	
CAC > 0	2.5898	1.1883	2.07	0.038	1.0536	6.3658
AVC > 0	2.2983	0.56316	3.40	0.001	1.4218	3.7152
1 SD increase of FRS	1.1526	0.13850	1.18	0.237	0.91073	1.4587
1 SD increase of hsCRP, mg/L	1.1188	0.09318	1.35	0.177	0.95036	1.3172
PEW (SGA > 1)	1.0998	0.2649	0.40	0.693	0.6859	1.7635
Ref LDKT (*n* = 159)						
DDKT (*n* = 81)	1.5629	0.56546	1.23	0.217	0.76907	3.1761
CKD G5D (*n* = 102)	2.7421	1.0082	2.74	0.006	1.3338	5.6373

Abbreviations: AVC, aortic valve calcium; CAC, coronary artery calcium; FRS, Framingham risk score; hsCRP, high sensitivity C‐reactive protein; HR, hazard ratio; PEW, protein‐energy wasting; SGA, subjective global assessment.

In patients with CAC > 400 AU, the hazard risk for CVE was 6.0 times higher (95% CI, 2.3–15.4, *p* < 0.001) compared to patients with no signs of CAC, after 6.4 years of follow‐up, independent of FRS at baseline and type of KFRT during follow‐up (CKD G5D, DDKT, and LDKT) (Table S2). The hazard ratio for all‐cause mortality was 7.4 (95% CI, 2.1–25.8, *p* = 0.002) in patients with CAC > 400 AU compared to patients without CAC (0 AU), independent of FRS and type of KFRT during follow‐up (Table S3). ROC curve analysis showed that CAC score 381 AU (*R*‐value = 0.80) was the best cut‐off in predicting CVE (Fig. S2), while a CAC score of 371 AU (*R*‐value = 0.84) was the best cut‐off to predict all‐cause mortality (Fig. S3).

### Medial calcification, CV events, and mortality

We included the 159 LDKT patients with CAC score in a larger LDKT cohort to investigate and compare the impact of MC compared to CAC. In this cohort of 230 LDKT patients, the median age was 46 years, 69% were males, 7% had diabetes, and 12% had CVD at time of KT (Table 3). Arterial biopsies for MC scoring were available in 200 of the LDKT patients, and in 173 patients CAC score was available. Although 126 patients (63%) had low‐grade MC, 74 patients (37%) presented with high‐grade MC. In the patients with low‐grade MC, the risk for CVE was 5.6% compared to 28.4% in patients with high‐grade MC (*p* < 0.001) after 6.4 years of follow‐up. The risk of death was also lower in patients with low‐grade MC: 1.6% versus 14.9% (*p* = 0.001) after 6.4 years of follow‐up. None of the patients experienced intra‐ or postoperative complications due to removal of part of the arteria epigastrica.

**Table 3:** Baseline clinical and biochemical characteristics in 230 living donor kidney transplantation (LDKT) patients according to degree of medial calcification in epigastric artery

	All	Low‐grade MC	High‐grade MC	No arterial biopsy	
	N = 230	N = 126	N = 74	N = 30	*p*‐Value
Age, years	46 (33–57)	40 (28–50)	51 (45–61)	50 (41–62)	<0.001
Male sex, *n* (%)	159 (69.1%)	71 (56.3%)	61 (82.4%)	27 (90.0%)	<0.001
Diabetes, *n* (%)	17 (7.4%)	0 (0.0%)	14 (18.9%)	3 (10.0%)	<0.001
CVD, *n* (%)	27 (11.7%)	7 (5.5%)	18 (24.3%)	2 (6.7%)	<0.001
Systolic BP, mm Hg	141 (130–155)	138 (128–152)	145(131–157)	152(133–166)	0.006
Diastolic BP, mm Hg	85 (76–93)	85 (78–93)	84 (74–92)	88 (80–98)	0.23
Framingham risk score	6.9 (3.2–14.7)	4.3 (1.8–8.5)	13.2 (5.7–22.4)	13.1 (7.3–18.4)	<0.001
Protein energy wasting (SGA > 1)	70 (30.4%)	47 (37.3%)	12 (16.2%)	11 (36.7%)	0.008
BMI, kg/m^2^	24.2 (22.3–26.5)	23.5 (21.3–25.9)	25.5 (23.7–27.8)	25.2 (23.2–27.5)	<0.001
Hand grip strength, % of normal	97.7 (79.6–109.3)	100.0 (81.5–109.3)	92.9 (74.4–111.6)	86.0 (79.1–110.5)	0.53
Hemoglobin, g/L	113 (105–121)	114 (104–121)	112 (106–121)	110 (104–128)	0.98
Albumin, g/L	35 (32–38)	35 (32–38)	35 (32–37)	33 (32–37)	0.17
Triglyceride, mmol/L	1.3 (1.0–1.9)	1.3 (0.9–1.9)	1.4 (1.1–1.9)	1.3 (0.9–2.0)	0.56
Total cholesterol, mmol/L	4.4 (3.6–5.1)	4.5 (3.9–5.2)	4.3 (3.4–5.0)	3.8 (3.0–4.8)	0.033
Calcium, mmol/L	2.3 (2.2–2.4)	2.3 (2.2–2.4)	2.3 (2.2–2.4)	2.2 (2.1–2.4)	0.23
Phosphate, mmol/L	1.7 (1.4–2.0)	1.7 (1.3–2.0)	1.6 (1.4–2.0)	1.6 (1.3–2.1)	0.97
iPTH, ng/L	260 (160–400)	234 (163–380)	309 (142–430)	270 (130–470)	0.47
hsCRP, mg/L	0.9 (0.3–2.2)	0.7 (0.3–2.0)	1.0 (0.5–2.4)	1.3 (0.6–2.6)	0.024
IL‐6, pg/ml	1.0 (0.5–2.0)	1.0 (0.4–1.9)	1.1 (0.5–1.8)	2.3 (1.8–4.1)	0.11
CAC score, AU	3 (0–152)	0 (0–33)	52 (7–975)	20 (0–293)	<0.001
AVC score, AU	0 (0–0)	0 (0–0)	0 (0–0)	0 (0–0)	0.022
All‐cause mortality, *n* (%)	16 (7.0%)	2 (1.6%)	11 (14.9%)	3 (10.0%)	0.001
CV events, *n* (%)	30 (13.0%)	7 (5.6%)	21 (28.4%)	2 (6.7%)	<0.001

*Note*: Data are presented as median (IQR, interquartile range) for continuous measures, and *n* (%) for categorical measures.

Abbreviations: AU, Agatston units; AVC, aortic valve calcium; BMI, body mass index; CAC, coronary artery calcium; CVD, cardiovascular disease; DBP, diastolic blood pressure; FRS, Framingham risk score; HDL, high‐density lipoprotein; %HGS, hand grip strength, converted to % of sex‐matched healthy controls; hsCRP, high sensitivity C‐reactive protein; IL‐6, interleukin‐6; iPTH, intact parathyroid hormone; LDL, low‐density lipoprotein; PEW, protein‐energy wasting; SBP, systolic blood pressure; SGA, subjective global assessment.

**Fig. 4:**
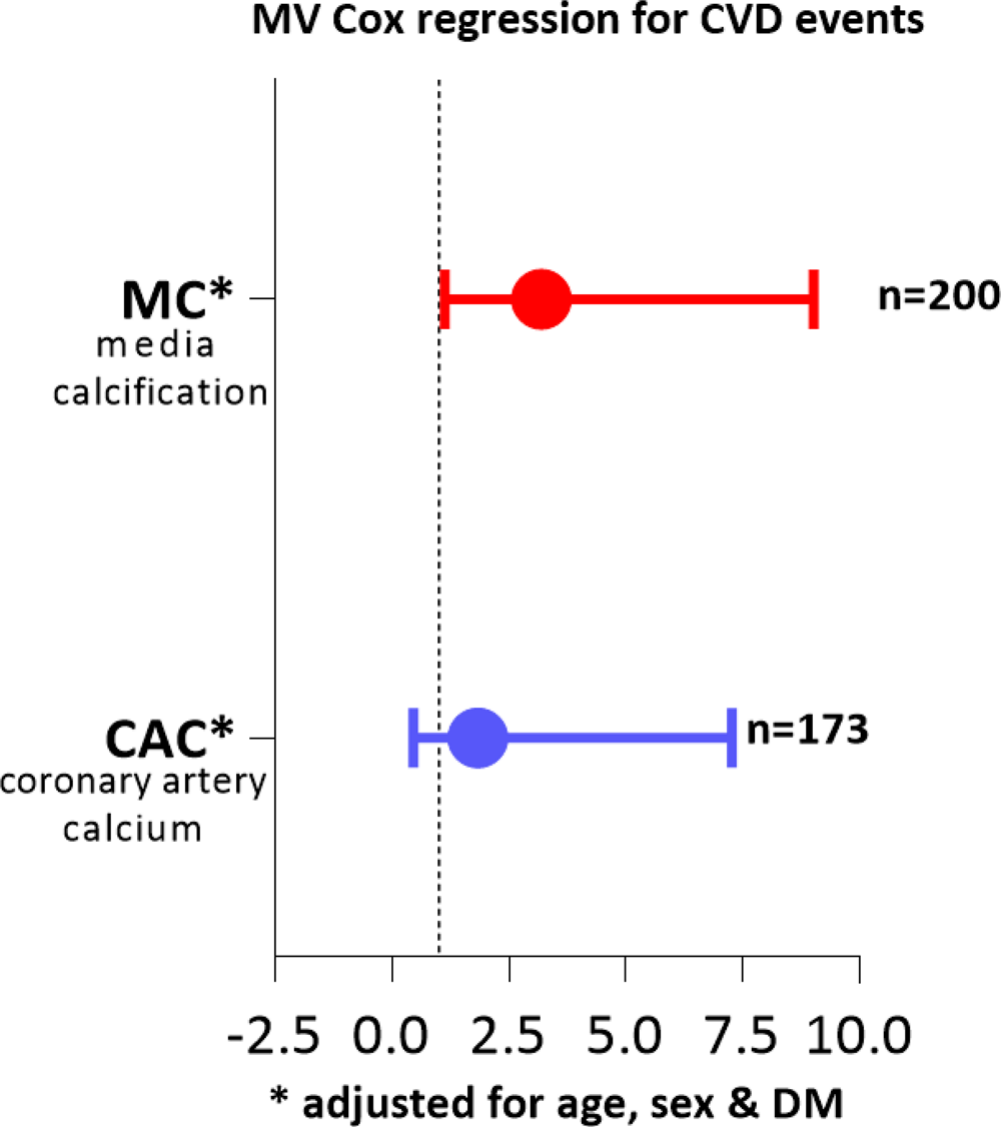
Multivariate Cox regression analysis of medial calcification [3.10 (1.12–9.02)] p < 0.05 and coronary artery calcium (CAC) [1.83 (0.46–7.28)] association with cardiovascular (CV) events in living donor kidney transplantation (LDKT).

Patients with high‐grade MC were older and more often male and diabetic. Moreover, they had a higher prevalence of CVD and PEW, higher systolic blood‐pressure, CAC, hsCRP, cholesterol, AVC, and FRS. Lower BMI may represent PEW (Table 3). The hazard ratio of CVE in patients with high‐grade MC was 3.1 compared to patients with low‐grade MC (95% CI, 1.12–9.02, *p* < 0.05) independent of age, sex, and diabetes mellitus at baseline (Fig. 4). In contrast, when comparing CAC score in a multivariate analysis with the same covariates, prevalent CAC score was not predictive of CVE during 6.4 years of follow‐up after LDKT (Fig. 4).

When the three KFRT groups (CKD G5D, DDKT, and LDKT) were compared, differences in age (median 67, 53, and 47 years, *p* < 0.001), diabetes (32%, 19%, and 8%, *p* < 0.001), CVD (35%, 16%, and 13%, *p* < 0.001), eGFR, BMI, and hand grip strength were evident (Table S1). In a Cox multivariate analysis of CVE, the hazard risk for CVE was 2.7 times higher in CKD G5D (HR 2.7, 95% CI, 1.3–5.5, *p* = 0.007) compared to LDKT independent of FRS and prevalent CAC at baseline (Table S2). The hazard risk of all‐cause mortality was higher in patients remaining on dialysis never receiving a KT (CKD G5D) during follow‐up (HR 33.6, 95% CI, 10.0–113.0, *p* < 0.001) independent of FRS and prevalent CAC at baseline (Table S3).

## Discussion

The main finding of our study is that presence of high‐grade medial calcification independently predicts a 3.1 times higher risk of CVE compared to low‐grade medial calcification after LDKT. Thus, classification of the degree of medial calcification in the arteria epigastrica by a pathologist offers similar or better prognostic information than a CT scan of coronary calcification (Fig. 4). This provides an opportunity to use this simple and inexpensive method to enhance prediction of CVE after KT in a group of low‐risk KFRT patients. As expected, median CAC score in this group of younger patients (less diabetes and lower prevalence of CVD) was significantly lower than in DDKT and CKD G5D. In a previous study [6], the epigastric artery was biopsied during KT in 41 mid‐age (45 ± 13 years) KT recipients (LDKT, *n* = 19; DDKT, *n* = 22). Our finding supports the observation in that study that medial calcification was found in 44% of the patients and that medial calcification was associated with diabetes and prevalent CVD. Unfortunately, that study did not include follow‐up data to evaluate the predictive value of medial calcification for future CVE.

We report that patients who remained on dialysis had a 33.6 times higher risk of mortality and a 2.7 times higher risk of CVE compared to LDKT patients, despite adjustment for FRS and CAC. Our observation that patients who remained on dialysis—that is, not eligible for KT, or accepted to the waitlist but not transplanted—had a 10‐year mortality approaching 100% supports previous studies of a mortality rate in dialysis patients close to 10 times higher than in the general population [29]. In order to prevent CKD patients from entering a state of progressive VC with severe CV complications that contraindicate KT, interventions to slow down the early vascular ageing process should be initiated at an early stage of the disease. Because novel drugs (such as inositol‐6‐phosphate) that inhibit the calcification process may within a couple of years be introduced for clinical treatment of ectopic calcification, a robust marker of the extent of arterial calcification is needed [30].

Our data demonstrate that the presence of high‐grade medial calcification at time of KT independently predicts future CVE. Although hypertension and kidney function improve after KT, multiple studies have shown that the progress of coronary and valvular calcification continues, but at a slower rate than in patients remaining on dialysis [31, 32, 33, 34, 35, 36]. Thus, pre‐existing medial calcification at the time of KT may serve as a nidus for continuous progression of ectopic calcification despite a lower metabolic risk factor profile after KT. Standard immunosuppressive protocols are associated with elevated risk for pro‐atherogenic conditions, such as posttransplant diabetes mellitus, dyslipidemia, and hypertension [37, 38]. Since the risk of CVE remains high in some—but not all—patients after KT, validated risk prediction tools are needed to identify KFRT patients with established medial calcification at the time of KT. The surgical procedure during KT (LD and DD) allows perioperative removal of a part of the arteria epigastrica (Fig. 2) without increased risk for the patient, so this may provide an opportunity to identify KT patients with a high risk of CVE and all‐cause mortality through scoring by a pathologist. Indeed, we show that in patients with low‐grade medial calcification at the time of KT, the risk of CVE during the observation period is low (5.6% vs. 28.4%).

The results of this study should be interpreted while taking several strengths and limitations into consideration. The careful phenotyping—including CT scans of the heart and biopsies from the arteria epigastrica—as well as the long observation period strengthens the study. Because there were no exclusion criteria, we reduced the risk of selection bias. There were no significant differences between included and excluded patients (Table 3). Our cohort was solely from the Stockholm region, but earlier national studies have not found any differences in patient survival or cause of death after KT between regions in Sweden. For these reasons, we believe the generalizability (external validity) is great. However, arteria epigastrica represent mid‐size muscular arteries, it should be acknowledged that the calcification process may differ in vascular beds of different size. Limitations also include the lack of matching between the three groups of KFRT patients. LDKT patients constituted a younger and healthier group of patients than DDLD and CKD G5D patients. Although ethical and practical reasons preclude arterial biopsy sampling in dialysis patients, arterial biopsies from the DDKT group would have benefited the study. The fact that a heart CT cannot differentiate between intimal and medial calcification also limits the study. It is notable that despite the comparatively healthy vascular phenotype in the younger group selected for LDKT (median CAC score 3 AU), as many as 37% exhibited high‐grade medial calcification when arteria epigastrica was graded. Thus, the risk of CVE after KT may be underestimated if the extent of calcification is based solely on a heart CT. Since biopsy of the arteria epigastrica is an invasive procedure, the scoring of medial calcification cannot be recommended as a risk score to be used prior to transplantation. However, it does identify patients at high risk for CVE, and it could be used to identify patients who should receive optimized preventive treatment.

In conclusion, scoring of medial arterial calcification predicts CVE and death after KT independently of recognized risk factors. As perioperative removal of part of the arteria epigastrica does not increase the risk for the patient during or after surgery, we suggest that this intraoperative procedure should be introduced and used in the standard clinical care of transplanted patients.

## Conflict of interest

Peter Stenvinkel serves on scientific advisory boards for AstraZeneca, Baxter Healthcare, Reata Pharmaceuticals, Vifor, and Fresenius Medical Care. Annette Bruchfeld reports personal fees from AstraZeneca, Bayer, ChemoCentryx, and Vifor.

## Author contribution

Helen Erlandsson designed the study, collected and analyzed data, and wrote and revised the manuscript. Abdul Rashid Qureshi, Jonaz Ripsweden, Magnus Söderberg, and Torkel B. Brismar provided analyzed data and revised the manuscript. Torbjörn Lundgren and Lars Wennberg collected arterial biopsies and revised the manuscript. Ida Haugen Löfman and Annette Bruchfeld revised the manuscript. Peter Stenvinkel designed the study, and completed and revised the manuscript.

## Supporting information


**Supplementary Table 1**: Baseline clinical and biochemical characteristics in 342 KFRT patients according to dialysis (CKD G5D), kidney transplantation with living donor (LDKT) and deceased donor (DDKT).
**Supplementary Table 2**: Multivariate Cox analysis for CV‐events in KFRT patients n=342, median follow‐up 6.4 years.
**Supplementary Table 3**: Multivariate Cox analysis for all‐cause mortality in KFRT patients n=342, median follow‐up 6.4 years.
**Supplementary Figure 1**: Classification of medial calcification.
**Supplementary Figure 2**: ROC: Area under curve (AUC) for CVD‐events of age and CAC‐score. Cut‐off level of CAC score was 381 AU (n=342).
**Supplementary Figure 3**: ROC: Area under curve (AUC) for All‐cause mortality of age and CAC‐score. Cut‐off level of CAC score was 371 AU (n=342).
