# Supplementary material for: Scoring of medial arterial calcification predicts cardiovascular events and mortality after kidney transplantation
Source: J Intern Med. 2022 Feb 11;291(6):813–23. doi: 10.1111/joim.13459 (PMC9306575; doi:10.1111/joim.13459)

Supplements tables and figures

Suppl Table 1 Baseline clinical and biochemical characteristics in 342 KFRT patients according to dialysis (CKD G5D), kidney transplantation with living donor (LDKT) and deceased donor (DDKT).

	Total<br>N=342	CKD G5D<br>N=102	LDKT<br>N=159	DDKT<br>N=81	p-value
Demography and clinical characteristics					
Age, years	53 (42-65)	67 (57-75)	47 (32-56)	53 (44-60)	<0.001
Male sex, n (%)	227 (66.4%)	67 (65.7%)	113 (71.1%)	47 (58.0%)	0.13
Diabetes, n (%)	55 (16.9%)	28 (32.2%)	12 (7.6%)	15 (18.8%)	<0.001
CVD, n (%)	64 (19.5%)	31 (35.2%)	20 (12.6%)	13 (16.0%)	<0.001
Systolic BP, mmHg	144 (130-157)	144 (132-165)	141 (129-156)	145 (130-154)	0.42
Diastolic BP, mmHg	85 (77-94)	82 (76-92)	86 (78-95)	88 (76-96)	0.11
eGFR epi ml/min	6.1 (5.1-7.8)	5.9 (4.9-7.6)	6.7 (5.4-8.9)	5.4 (4.6-6.9)	<0.001
PEW (SGA>1)	119 (36.5%)	37 (41.1%)	50 (31.6%)	32 (41.0%)	0.21
BMI, kg/m^2	24.8 (22.6-27.8)	25.7 (23.1-29.1)	24.3 (22.4-26.9)	24.8 (22.2-27.2)	0.041
%HGS	85.6 (69.8-102.3)	72.1 (62.8-83.7)	97.7 (80.8-109.3)	86.0 (70.4-97.7)	<0.001
Hemoglobin, g/L	112 (104-120)	114 (102-121)	112.5 (104-121)	110 (104-116)	0.30
Albumin, g/L	34 (31-37)	32 (28-35)	35 (32-38)	34 (31-37)	<0.001
Triglyceride, mmol/L	1.5 (1.1-2.1)	1.6 (1.2-2.2)	1.3 (1.0-1.8)	1.7 (1.3-2.4)	<0.001
Total cholesterol, mmol/L	4.4 (3.7-5.2)	4.7 (3.8-5.3)	4.3 (3.6-5.1)	4.5 (3.7-5.3)	0.11
Calcium, mmol/L	2.3 (2.1-2.4)	2.3 (2.1-2.4)	2.3 (2.1-2.4)	2.3 (2.2-2.4)	0.12
Phosphate, mmol/L	1.8 (1.5-2.1)	1.8 (1.5-2.2)	1.7 (1.4-2.0)	2.0 (1.6-2.2)	0.002
iPTH, ng/L	259 (160-426)	245 (117-396)	262 (167-415)	275 (160-457)	0.40
hsCRP, mg/L	1.4 (0.7-4.2)	3.8 (1.2-8.1)	0.9 (0.4-2.2)	1.8 (1.0-7.0)	<0.001
IL-6, pg/mL	3.5 (1.7-7.4)	6.6 (4.6-10.2)	1.1 (0.4-2.1)	3.2 (2.2-7.4)	<0.001
CAC score, AU	74 (0-872)	847 (306-2168)	3 (0-150)	65 (0-422)	<0.001

Data are presented as median (IQR, interquartile range) for continuous measures, and n (%) for categorical measures;
Abbreviations: AVC, aortic valve calcium; CVD, cardiovascular disease; SBP, systolic blood pressure; DBP, diastolic blood pressure; FRS, Framingham CVD risk score; PEW, protein-energy wasting; SGA, subjective global assessment; BMI, body mass index; %HGS, hand grip strength, converted to % of sex-matched healthy controls; HDL, high-density lipoprotein; LDL, low-density lipoprotein; iPTH, intact parathyroid hormone; hsCRP, high sensitivity C-reactive protein; IL-6, interleukin-6; AU, Agatston units; CAC, coronary artery calcium; ACEi/ARB, angiotensin-converting enzyme inhibitor/ angiotensin II receptor blockers

Suppl Table 2 Multivariate Cox analysis for CV-events in KFRT patients n=342, median follow-up 6.4 years.

_t	Haz. ratio	Std err.	z	P>|z|	[95% conf.	interval]
Ref CAC <0 (n=111)						
CAC 1-200 (n=87)	1.9345	.98913	1.29	0.197	.71013	5.2698
CAC 201-400 (n=24)	2.2600	1.4856	1.24	0.215	.62315	8.1967
**CAC >401** (n=120)	**5.9775**	**2.8835**	**3.71**	**0.000**	**2.3222**	**15.386**
1-SD of Framingham CVD risk score	1.0462	.12856	0.37	0.713	.82235	1.3312
Ref LDKT (n=159)						
DDKT (n=81)	1.6049	.57689	1.32	0.188	.79339	3.2465
**CKD G5D** (n=102)	**2.6711**	**.97381**	**2.69**	**0.007**	**1.3072**	**5.4577**

Suppl Table 3 Multivariate Cox analysis for all-cause mortality in KFRT patients n=342, median follow-up 6.4 years.

_t	Haz. ratio	Std err.	z	P>|z|	[95% conf. interval]	
Ref CAC <0 (n=111)						
CAC 1-200 (n=87)	2.4164	1.6280	1.31	0.190	.6451	9.0501
**CAC 201-400 (n=24)**	**5.4253**	**3.8915**	**2.36**	**0.018**	**1.3300**	**22.1303**
**CAC >401 (n=120)**	**7.4257**	**4.7238**	**3.15**	**0.002**	**2.1343**	**25.8358**
1-SD of Framingham CVD risk score	.89122	.10489	- 0.98	0.328	.70761	1.1224
Ref LDKT (n=159)						
DDKT (n=81)	3.3218	2.1607	1.85	0.065	.9283	11.8863
**CKD G5D (n=102)**	**33.6522**	**20.8062**	**5.69**	**0.000**	**10.0170**	**113.0543**

Suppl Fig 1 Classification of medial calcification

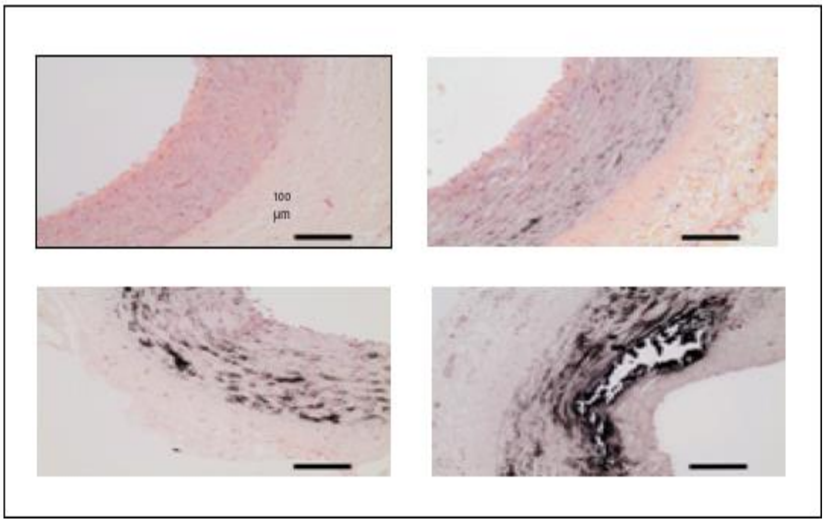

Figure legend. Example images of typical score 0 (top left), score 1 (top right), score 2 (bottom left) and score 3 (bottom right) lesions. Calcium phosphate deposits are visualized in black using the von Kossa histochemical stain. Scale bar represents 300 µm.

Suppl Fig 2 ROC: Area under curve (AUC) for CVD-events of age and CAC-score. Cut-off level of CAC score was 381 AU (n=342).

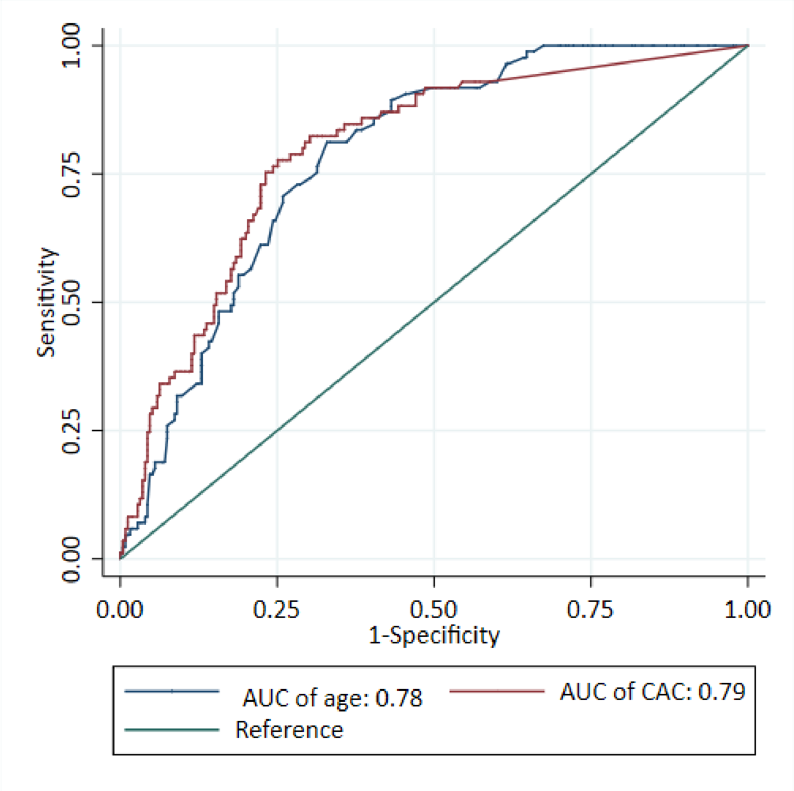

Suppl fig 3 ROC: Area under curve (AUC) for All-cause mortality of age and CAC-score. Cut-off level of CAC score was 371 AU (n=342)

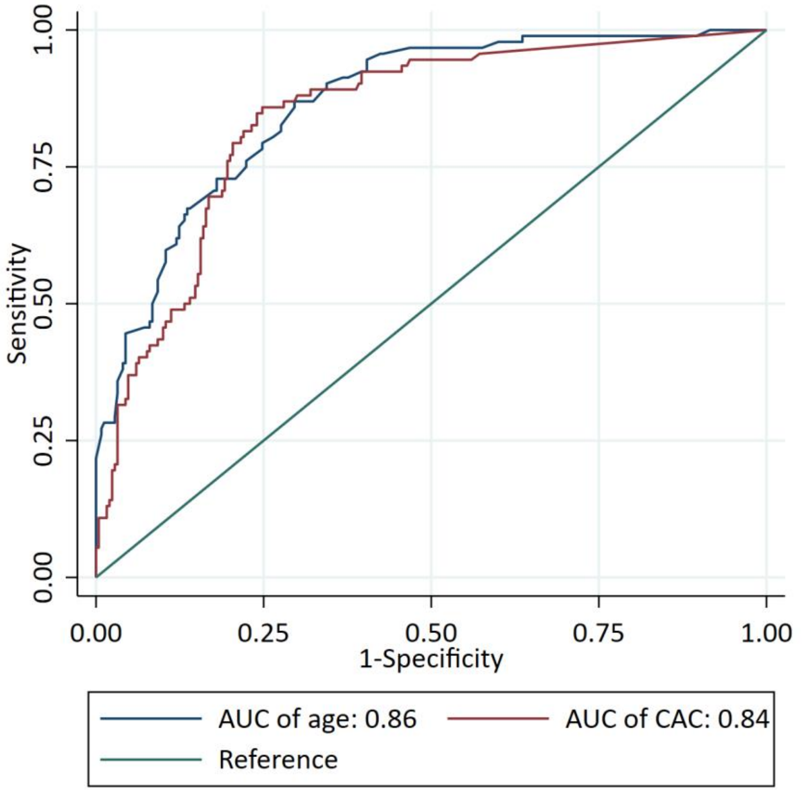

Supplement: Supplementary file 1 — Supplementary Table 1: Baseline clinical and biochemical characteristics in 342 KFRT patients according to dialysis (CKD G5D), kidney transplantation with living donor (LDKT) and deceased donor (DDKT). Supplementary Table 2: Multivariate Cox analysis for CV‐events in KFRT patients n=342, median follow‐up 6.4 years. Supplementary Table 3: Multivariate Cox analysis for all‐cause mortality in KFRT patients n=342, median follow‐up 6.4 years. Supplementary Figure 1: Classification of medial calcification. Supplementary Figure 2: ROC: Area under curve (AUC) for CVD‐events of age and CAC‐score. Cut‐off level of CAC score was 381 AU (n=342). Supplementary Figure 3: ROC: Area under curve (AUC) for All‐cause mortality of age and CAC‐score. Cut‐off level of CAC score was 371 AU (n=342). [file JOIM-291-813-s001.pdf]
